# Supplementary material for: Drug repurposing based on a quantum-inspired method versus classical fingerprinting uncovers potential antivirals against SARS-CoV-2
Source: PLoS Comput Biol. 2022 Jul 18;18(7):e1010330. doi: 10.1371/journal.pcbi.1010330 (PMC9333455; doi:10.1371/journal.pcbi.1010330)
Supplement: S1 Fig — The relative IC50 was calculated based on Fig 6 data. The relative IC50 was established as the concentration required to bring the curve down to the middle point between the top and bottom plateaus of the curve. (PDF) [file pcbi.1010330.s001.pdf]

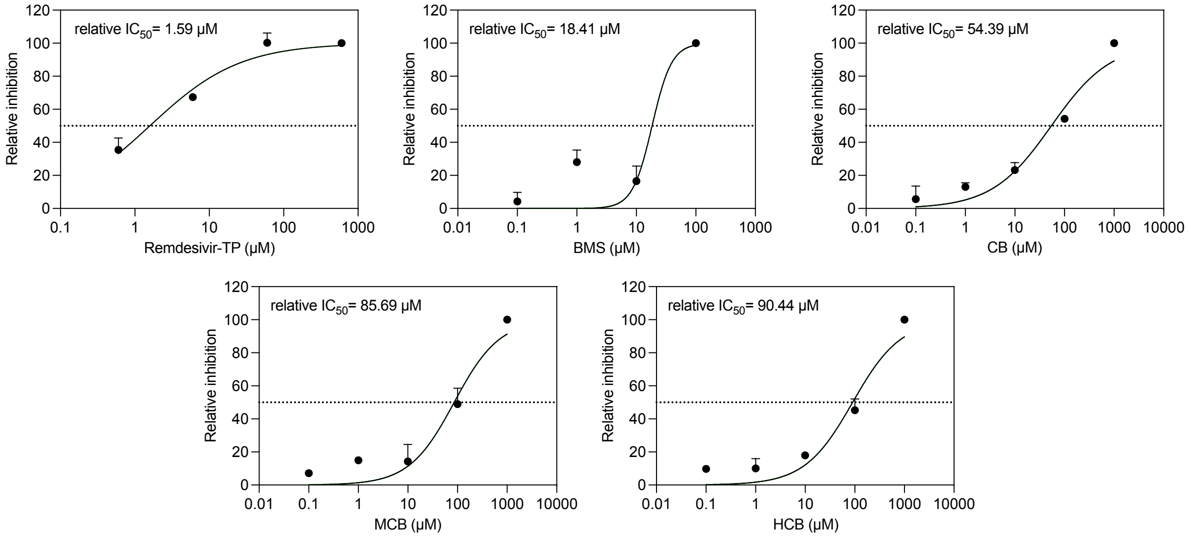


**S1 Fig. *In vitro* relative $IC_{50}$ of predicted compounds**. The relative $IC_{50}$ was calculated base don Fig 6 data as the concentration required to bring the curve down to the middle point between the top and bottom plateaus of the curve.
