# Supplementary material for: Drug repurposing based on a quantum-inspired method versus classical fingerprinting uncovers potential antivirals against SARS-CoV-2
Source: PLoS Comput Biol. 2022 Jul 18;18(7):e1010330. doi: 10.1371/journal.pcbi.1010330 (PMC9333455; doi:10.1371/journal.pcbi.1010330)
Supplement: Attachment — Submitted filename: Response_to_Reviewers.pdf [file pcbi.1010330.s002.pdf]

School of Immunology and Microbial Sciences
King's College London

Asthma UK Centre in Allergic Mechanisms of Asthma

Dr Rocio T Martinez-Nunez
Dept. Infectious Diseases
5th Floor Tower Wing | Guy's Campus
King's College London
London SE1 9RT
Tel: +44(0)2071880595
rocio.martinez_nunez@kcl.ac.uk

Comments to the Authors:
Please note here if the review is uploaded as an attachment.
We have uploaded the revised versions (clean and marked-up), a point-by-point response and a cover letter.

Reviewer #1: Jimenez-Guardeño and al. have implemented computational structural modeling to repurpose compounds structurally similar to Ramdesivir, the only current antiviral FDA-approved compound for the treatment of severe COVID-19. The aim of the study is to identify structurally similar analog, clinically available, that could overcome emerging limits for Ramdesivir, such as multiple side effects and costs-related issues. The authors claim that, indeed, there is an urgent need to identify novel antiviral compounds that exhibit low to no side effects, and that are readily and economically available. To this aim, the authors implemented both novel and traditional computing approaches for handling complex information such as 3D structures to identify structurally similar analogs. The two methods yielded different compounds, with some overlap, and predicted, among others, different forms of cobalamin, also known as vitamin B12, as best candidates. Among others, the authors focused on assessing the effect of different concentrations of vitamin B12 forms on SARS-CoV-2 infection of two different cell lines and demonstrated that vitamin B12 forms were effective at inhibiting replication of all variants of SARS-CoV-2 assessed, namely England 2 (England 02/2020/407073), B.1.1.7 (Alpha), B.1.351 (Beta) and 55 B.1.617.2 (Delta).

Overall, this is an interesting, well-written manuscript, whose results have the potential to support further preclinical and clinical research to repurpose vitamin B12 forms against SARS CoV-2 infection and variants.

The computational pipeline and preclinical validation are well presented. However, this reviewer finds there is a lack of mechanistic demonstration about the effective similarity results and about the mode of action of vitamin B12 forms compared to Ramdesivir. In the current form, the similarity indeed is based on results from computational modeling (see tables and Fig1 c-d) only. A more robust, mechanistic demonstration could result, as for example, by investigating competitive or affinity binding analysis against the natural (expected) target, i.e. RNA-dependent RNA polymerase (RdRp) enzyme or, alternatively, by demonstrating the effective inhibition of RNA polymerization activity as demonstrated for Ramdesivir (PMID: 32358203). Competitive/comparative studies between Ramdesivir and will vitamin B12 forms will be also of advantage. Adding this data will provide this study with a mechanistic demonstration about the relevant target and potential antiviral mechanism of vitamin B12 forms; in contrast, in the absence of such studies, one cannot role-out that vitamin B12 forms might target another cellular/molecular mechanisms inhibiting SARS-CoV-2, regardless the structural similarity with Ramdesivir. Adding this data will support the effectiveness of the modeling approach and will guide additional investigation of vitamin B12 forms as antiviral drugs based on a well-described Mode of Action.

We thank the Reviewer for their recommendation; we agree that an *in vitro* approach measuring the effect of the different compounds on RdRP activity would further support our modelling and cellular data. In the new Fig 6 we employed an *in vitro* polymerization assay that only included RdRp. Our data showed that BMS and different forms of vitamin B12 inhibited the polymerase activity of RdRP in isolation, confirming that it is a direct molecular target of these compounds. Although potential additional effects on cells cannot be excluded

from Figures 2-5, the data in Figure 6 indeed demonstrate that BMS and vitamin B12 forms directly inhibit the RNA polymerase activity of SARS-CoV-2 RdRP.

Reviewer #2: In this manuscript, the authors used computational methods to search for known drugs that share similarity to Remdesivir, the only approved antiviral against SARSCoV-2. For the search, they used a Quadratic Unbounded Binary Optimization (QUBO) model run on a "quantum-inspired device", and the traditional Tanimoto fingerprint model. The searches identified a number of hits, including multiple variants of vitamin B12. These hits were tested for growth-inhibitory and cytotoxic effects in cell culture models of SARS-CoV-2, and for effect in inhibiting the replication of various strains of SARS-CoV-2. The results show that the hits inhibit cell growth and prevent the replication of SARS-CoV-2, albeit at very high concentrations (BMS = 30uM; cobamamide = 500uM; methylcobalamin = 500uM; hydroxocobalamin = 500uM). Overall, while the final findings themselves are not particularly transformative, the manuscript describes a set of interesting results from well-executed calculations and experiments that are of potential relevance to a cross-section of PLoS Comput Biol readers. I therefore recommend publication after a revision addressing the following significant concerns, mostly related to presentation.

BMS is cytotoxic (Fig 2) and therefore not useful as a therapeutic agent. The cobalamin variants are non-toxic and might be tolerated at high concentrations. However, an IC50 close to 500uM suggests a roughly 100mg/kg administration for any therapeutic benefit. This seems way too high even for a completely non-toxic and well-behaved agent. The authors provided some arguments to suggest that B12 may have an antiviral therapeutic value if administered at high dose, perhaps administered in a way that it is localized only in the airways. This is not convincing. In this reviewer's view, it is important to acknowledge the unlikeliness of the compounds being used to treat COVID patients (there is enough misleading information in the literature regarding therapies for COVID patients). Instead, one could discuss the potential of the hits to serve as starting points for rational design of new inhibitors/derivatives.

We thank the Reviewer for their comment, and we agree that it is very important to avoid the potential use of our data in certain misleading fora. We have modified the text to clarify this important point. As an example, we have added in Discussion ‘However, pharmacodynamics and different absorption of vitamin B12 at these theoretically required high doses make it an unlikely therapy’.

Another (related) concern regarding the message of the manuscript is the emphasis on QUBO/quantum. For example, the concluding sentence in Abstract states "Our quantum-inspired screening method can be employed in future searches for novel pharmacologic inhibitors, thus providing an approach for accelerating drug deployment." However, this approach did not deliver better results than the simpler, faster and traditional Tanimoto fingerprint model. The two models predicted the same compound as their top hit. They differed in their second-best hit, but the B12 compounds -- a major focus of the paper—were predicted as second best by the Tanimoto model. So why wouldn't I be just happy using Tanimoto? Therefore, here, too, a more balanced and nuanced presentation would seem to be in order.

Thank you for this suggestion. We have clarified in the text that we employed Tanimoto as a benchmarked validated approach, while QUBO produced similar results only in the top

compound. We further enhance the relevance of QUBO, which we believe our data validate given its predictions and experiments with BMS, and the potential use of QUBO in other disciplines or more complex comparisons, where the high computational demand is more fit for a quantum-inspired device.
